# Efficacy of the Low Dose Apatinib plus Chemotherapy on Advanced Gastric Carcinoma

**DOI:** 10.1155/2022/3009494

**Published:** 2022-03-30

**Authors:** Shen Gao, Xuan Li, Weigang Shi, Limin Huo, Huimin Liu

**Affiliations:** ^1^Department of General Surgery, Handan First Hospital, Handan, Hebei Province, China; ^2^Handan First Hospital, Handan, Hebei Province, China; ^3^Affiliated Hospital of Hebei Engineering University, Handan, Hebei Province, China

## Abstract

**Objective:**

To evaluate the efficacy of low dose apatinib plus chemotherapy on advanced gastric carcinoma.

**Methods:**

Eligible 50 patients with advanced gastric carcinoma admitted to the hospital from January 2019 to March 2020 were enrolled, and they were assigned into the control group (*n* = 25, chemotherapy) and observation group (apatinib plus chemotherapy). Changes of CEA, CA72-4, and VEGF levels were measured, and the efficacy of the two groups was evaluated by referring to KPS and RECIST.

**Results:**

Significant reduction was observed in CEA, CA72-4, and VEGF in both groups, and the treatment in the observation group resulted in a greater reduction (all *P* < 0.05). The observation group obtained significantly higher KPS scores of compared with the control group (*P* < 0.05). In addition, the treatment in the observation group led to a better control rate in relative to the control group according to RECIST the score (*P* < 0.05).

**Conclusion:**

The combination of low dose apatinib and chemotherapy might be a promising option for advanced gastric cancer and it merits clinical application.

## 1. Introduction

As one of the top five major malignant tumors worldwide, gastric cancer exhibits a high incidence and fatality rate, and threatens human health [1–3]. Currently, the mainstays for gastric cancer include conventional chemotherapy, radiotherapy, and surgery. However, for some advanced patients, the results of these methods are not satisfying [4–6]. With this background, massive studies about apatinib combined with chemotherapy are ongoing. Apatinib alone as second-line or followed therapy in treating metastatic adenocarcinoma gastric cancer showed improved progression-free survival (PFS) and overall survival. Preclinical and clinical studies indicated that antiangiogenic therapy improved the efficacy of immune checkpoint inhibitors. Apatinib selectively inhibits vascular endothelial growth factor (VEGF) receptor (VEGFR) 2 and showed activity in advanced cancer in retrospective reports [7–9]. Nevertheless, no study has yet specifically determined the efficacy of chemotherapy and apatinib in advanced gastric cancers. To fill the gap, we intended to evaluate its therapeutic effect aiming to provide a certain reference for clinical application.

## 2. Methods

### 2.1. Study Design and Participants

Between January 2019 and March 2020, 50 patients with advanced gastric cancer treated in our hospital were enrolled and were divided into two groups. The baseline data were well balanced with respect to the age, weight, clinical stage, and CEA, CA72-4, and VEGF levels between the two groups before enrollment, as shown in Table 1. The protocol was approved by the ethics committee of Affiliated Hospital of Hebei Engineering University (No. HBEUAH3987). All subjects gave written informed consent in accordance with the Declaration of Helsinki.

### 2.2. Inclusion and Exclusion Criteria

Inclusion criteria were as follows: (1) patients confirmed to be gastric cancer disease judgment, (2) patients who have failed in general treatment, (3) patients with expected survival more than three months, (4) patients with sound liver and kidney functions, (5) patients with no complications that would affect the results of this experiment, and (6) patients who voluntarily signed informed consent.

Exclusion criteria were as follows: (1) patients with hypertension, (2) patients with cardiopulmonary dysfunction, (3) patients who do not cooperate with clinical follow-up, and (4) patients with incomplete clinical data.

### 2.3. Treatment Methods

Control group: patients received FOLFOX4 regimen and chemotherapy for two weeks. (1) oxaliplatin (Shenzhen Neptunus Drug manufacturing company, SFDA approval No. H20031048, 85 mg/m^2^) was intravenously administered for 2 h, on day 1; (2) calcium folinate (Henan Furen Huaiqingtang Pharmaceutical enterprise, SFDA approval No. H20084204, 200 mg/m^2^) was intravenously administered for 2h, on day 1; (3) 5-fluorouracil (Hainan Sinochem United Pharmaceutical Industry Co., Ltd., SFDA approval number H20051626, 400 mg/m^2^) was intravenously administered on day 1; and (4) 5-fluorouracil (600 mg/m^2^) was intravenously administered for 22 h, on day 1 and day 2.

Observation group: patients received 500 mg of apatinib (Jiangsu Hengrui Pharmaceutical enterprise, SFDA approval No. H20140105) orally every day on the basis of chemotherapy regimen in the control group [10].

### 2.4. Outcome Measures

Tumor markers include carcinoembryonic antigen (CEA), CA72-4, and vascular epidermal growth factor (VEGF). Serum free VEGF was measured by ELISA, the kit was produced by Chemicon Company in the United States, and the operation was carried out according to the instructions. Roche 2010 electrochemiluminescence instrument was used to determine CEA and CA724, and the instrument supporting reagents were used.

The effectiveness was assessed by referring to Response Evaluation Criteria In Solid Tumors (RECIST). Complete response (CR) was as follows: disappearance of all target lesions. Any pathological lymph nodes (whether target or nontarget) must have reduction in short axis to <10 mm. Partial response (PR) was as follows: at least a 30% decrease in the sum of diameters of target lesions, taking as reference the baseline sum diameters. Progressive disease (PD) was as follows: at least a 20% increase in the sum of diameters of target lesions, taking as reference the smallest sum on study (this includes the baseline sum if that is the smallest on study). In addition to the relative increase of 20%, the sum must also demonstrate an absolute increase of at least 5 mm (note: the appearance of one or more new lesions is also considered progression). Stable disease (SD) was as follows: neither sufficient shrinkage to qualify for PR nor sufficient increase to qualify for PD, taking as reference the smallest sum diameters while on study.

The physical health was evaluated using Karnofsky performance status (KPS). The KPSS scale ranges from 100, which implies full functional capability to carry out normal daily activities without clinical evidence (symptoms or signs) of disease, to zero, which implies death. Significant intervening scores include 70 (cares for self (toileting, feeding, bathing, and dressing), but unable to carry out normal activity or do active work such as housekeeping, school activities, and driving a car), 50 (requires considerable assistance and frequent medical care), 40 (disabled: requires special care and assistance, in bed more than 50% of time), 30 (severely disabled: hospitalization necessary; active supportive treatment necessary, almost always in bed), 20 (very sick: hospitalization necessary and requires extensive nursing care by professionals and (or family)), and 10 (moribund, fatal processes progressing rapidly).

### 2.5. Statistical Analysis

All data analysis was performed with SPSS 20.0. Count data and measurement data were verified via *χ*^2^ and *t*-test and presented in the form of cases (percentage) and (mean ± standard deviation). All data were tested at a significance level of .05 (2-sided).

## 3. Results

### 3.1. Comparison of CEA ,CA72-4, and VEGF

**Figure 1:**
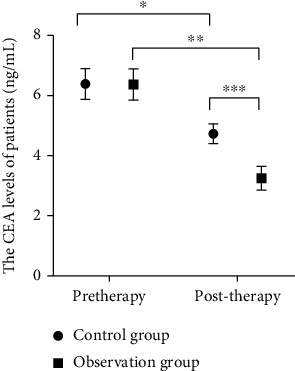
CEA expression levels of the two groups of patients before and after treatment. Note: CEA: carcinoembryonic antigen; *X*: before and after treatment; *Y*: CEA levels of the two groups of patients. The CEA levels of patients in control group between treatment were (6.39 ± 0.51 ng/mL) and (4.37 ± 0.33 ng/mL), while the CEA levels of patients of the observation group were (6.37 ± 0.35 ng/mL) and (3.25 ± 0.39 ng/mL) before and after treatment. ^∗^ and ^∗∗^ indicate a significant difference of CEA between all patients before and after treatment (control group: *t* = 16.17, *P* < 0.01; observation group: *t* = 28.40, *P* < 0.01). ^∗∗∗^ indicates that there is a significant difference in CEA levels between all patients after treatment (*t* = 17.14, *P* < 0.01).

**Figure 2:**
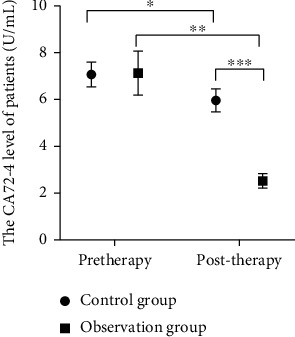
CA72-4 expression levels of all groups of patients between treatment. Note: CA72-4: cancer antigen 72-4; *X*: before and after treatment; *Y*: CA72-4 levels of the two groups of patients. The levels of CA72-4 between treatment in the control group were (7.07 ± 0.53 U/mL) and (5.96 ± 0.49 U/mL), while the levels of CA72-4 in the observation group before and after treatment were (7.13 ± 0.94 U/mL) and (2.52 ± 0.31 U/mL). ^∗^ and ^∗∗^ indicate a significant difference in levels of CA72-4 between all groups of patients before and after treatment (control group: *t* = 7.69, *P* < 0.01; observation group: *t* = 23.29, *P* < 0.01). ^∗∗∗^ indicates that there is a difference in levels of CA72-4 between all groups after treatment (*t* = 29.66, *P* < 0.01).

**Figure 3:**
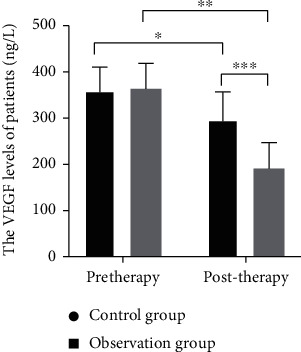
VEGF expression levels of all groups of patients between treatment. Note: VEGF: vascular epidermal growth factor; *X*: before and after treatment; *Y*: the expression level of VEGF before and after treatment in the two groups. The VEGF levels of patients in control group between treatment were (395.3 ± 51.12 ng/L) and (297 ± 60.03 ng/L), while the VEGF levels of patients of observation group were (363.3 ± 52.34 ng/L) and (191 ± 56.31 ng/L). ^∗^ and ^∗∗^ indicate a significant difference in VEGF levels between all patients between treatment (control group: *t* = 3.95, *P* < 0.01; observation group: *t* = 10.91, *P* < 0.01). ^∗∗∗^ indicates that there is a significant difference in VEGF levels between all groups of patients after treatment (*t* = 6.44, *P* < 0.01).

Significant reduction was observed in CEA, CA72-4, and VEGF in both groups, and the treatment in the observation group resulted in a greater reduction (all *P* < 0.05, Figures 1–3).

### 3.2. Comparison of KPS Scores

**Figure 4:**
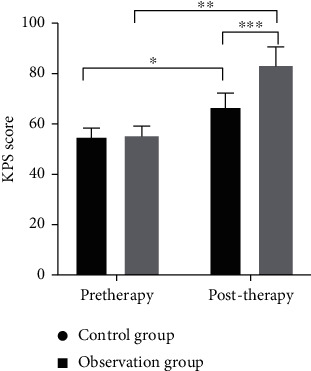
KPS scores of all groups of patients between treatment. Note: KPS: Karnofsky performance status; *X*: before and after treatment. *Y*: KPS scores of the two groups of patients before and after treatment. The KPS scores of the control group before and after treatment were (55.27 ± 3.09) and (67.00 ± 5.30), while the KPS scores of the observation group were (55.09 ± 4.11) and (83.00 ± 7.63) before and after treatment. ^∗^ and ^∗∗^ indicate that the KPS score levels of all groups of patients between treatment have significant differences (control group: *t* = 9.56, *P* < 0.01; observation group: *t* = 16.10, *P* < 0.01). ^∗∗∗^ indicates that there is a significant difference in the KPS scores of all groups of patients after treatment (*t* = 8.61, *P* < 0.01).

The observation group obtained significantly higher KPS scores of compared with the control group (*P* < 0.05, Figure 4).

### 3.3. Comparison of Treatment Effects

The treatment in the observation group led to a better control rate in relative to the control group according to RECIST the score (*P* < 0.05, Table 2).

## 4. Conclusion

At present, the treatment of advanced gastric cancer mostly uses second-line chemotherapy to inhibit the patient's lesions [10, 11]. However, studies have shown that although chemotherapy can prolong the life cycle of patients to a certain extent, it is still difficult to achieve the expected therapeutic effect [12, 13]. Therefore, a combination of targeted drugs and chemotherapy is often used to intervene in patients' lesions in clinical practice. In this study, we analyzed the clinical application value of combination therapy by comparing the efficacy of monotherapy and apatinib in combination with chemotherapy.

In this study, compared with receiving a single chemotherapy intervention, patients receiving apatinib targeted therapy on this basis exhibited a marked lower level of certain tumor markers. Apatinib is a new type of small molecule targeting protein that can effectively inhibit the formation of blood vessels around cancer tissues, thereby effectively inhibiting the invasion and migration of gastric cancer cells [14]. A previous study pointed out that the level of VEGF in the patient's serum is significantly correlated with the clinical stage of the tumor [15]. According to our results, the serum VEGF level of patients decreased significantly after treatment in patients who suffer from advanced gastric cancer. VEGFR facilitates the proliferation of vascular endothelial cells by activating the MAPK signaling pathway. The malignant expansion of tumors usually requires a lot of energy. As its scale continues to expand, cancer cells need to secrete growth factors such as VEGF, EGF, and ECF to promote the formation of blood vessels around them, thereby increasing the efficiency of nutrient transport [16]. The formation of blood vessels around the tumor is not only conducive to its own proliferation, but also provides favorable conditions for its metastasis and spread [17]. CEA and CA72-4 are also important factors that reflect the condition of cancer patients clinically. In this study, compared with single chemotherapy, the levels of CEA and CA72-4 witnessed a significantly more reduction after receiving apatinib treatment. All these might be attributed to the fact that apatinib, as an oral small molecule TKI against angiogenesis, can block VEGFR-2 in advanced GCA patients and reduce the activation of mitogen-activated protein kinase, thus inhibiting the proliferation of vascular endothelial cells. Preliminary studies reported a promising result of apatinib treatment for patients with advanced rectal cancer, advanced non-small-cell lung cancer, and advanced cervical cancer, in which their serum CEA levels decreased significantly, the survival time prolonged, with good safety profile [18–21]. Nevertheless, there is no consensus about a first-line chemotherapy regimen for patients with advanced gastric cancer, and this study thus was designed to intend to specifically determine an effective therapy.

Although our study leads the way in exploring the therapy for gastric cancer, certain limitations merit attention. The small sample size should be stated as a major limitation of this study. It is suggested that future trials be planned with larger sample size to further verify the efficacy and safety of therapy.

## Figures and Tables

**Table 1:** Comparison of general data.

Groups	Age (years)	Body weight	Male/female	Clinical staging	Stage IV
Control group	57.20 ± 2.10	62.19 ± 4.70	3 : 2	9	16
Observation group	56.90 ± 2.30	62.30 ± 4.60	3 : 2	7	18
*t*/*X*^2^	0.48	0.08	0	0.36	
*P*	*P* > 0.05	*P* > 0.05	*P* > 0.05	*P* > 0.05	

**Table 2:** Comparison of the therapeutic effects of different programs on patients (*n* (%)).

Groups	*n*	Therapeutic effects				Control rate
		CR	PR	SD	PD	
Control group	25	0 (0%)	3 (12%)	8 (32%)	14 (56%)	(12) 44%
Observation group	25	0 (0%)	9 (36%)	11 (44%)	5 (20%)	(20) 80%
*X* ^2^						6.88
*P*						<0.01

Note: CR: complete response; PR: partial response; SD: stable disease; PD: progressive disease.

## Data Availability

The datasets used during the present study are available from the corresponding authors upon reasonable request.
